# FHL2 Genetic Polymorphisms and Pro-Diabetogenic Lipid Profile in the Multiethnic HELIUS Cohort

**DOI:** 10.3390/ijms24054332

**Published:** 2023-02-22

**Authors:** Jayron J. Habibe, Ulrika Boulund, Maria P. Clemente-Olivo, Carlie J. M. de Vries, Etto C. Eringa, Max Nieuwdorp, Bart Ferwerda, Koos Zwinderman, Bert-Jan H. van den Born, Henrike Galenkamp, Daniel H. van Raalte

**Affiliations:** 1 Department of Medical Biochemistry, Amsterdam UMC, Amsterdam Cardiovascular Sciences, and Amsterdam Gastroenterology, Endocrinology and Metabolism, University of Amsterdam, 1105 AZ Amsterdam, The Netherlands; 2 Department of Physiology, Amsterdam UMC, Vrije Universiteit Medical Center, 1081 HV Amsterdam, The Netherlands; 3 Amsterdam Cardiovascular Sciences, and Amsterdam Gastroenterology, Endocrinology and Metabolism, University of Amsterdam, 1081 HZ Amsterdam, The Netherlands; 4 Department of Experimental Vascular Medicine, Amsterdam UMC, University of Amsterdam, 1105 AZ Amsterdam, The Netherlands; 5 Department of Physiology, Cardiovascular Institute Maastricht, 6229 ER Maastricht, The Netherlands; 6 Department of Clinical Epidemiology, Amsterdam UMC, University of Amsterdam, 1105 AZ Amsterdam, The Netherlands; 7 Department of Internal Medicine, Amsterdam UMC, University of Amsterdam, 1105 AZ Amsterdam, The Netherlands; 8 Department of Public and Occupational Health, Amsterdam UMC, University of Amsterdam, 1105 AZ Amsterdam, The Netherlands; 9 Department of Internal Medicine, Diabetes Center, Amsterdam UMC, 1081 HV Amsterdam, The Netherlands

**Keywords:** polymorphism, LDL-C, dyslipidemia, TG, cohort study, HELIUS, FHL2

## Abstract

Type 2 diabetes mellitus (T2D) is a prevalent disease often accompanied by the occurrence of dyslipidemia. Four and a half LIM domains 2 (FHL2) is a scaffolding protein, whose involvement in metabolic disease has recently been demonstrated. The association of human FHL2 with T2D and dyslipidemia in a multiethnic setting is unknown. Therefore, we used the large multiethnic Amsterdam-based Healthy Life in an Urban Setting (HELIUS) cohort to investigate FHL2 genetic loci and their potential role in T2D and dyslipidemia. Baseline data of 10,056 participants from the HELIUS study were available for analysis. The HELIUS study contained individuals of European Dutch, South Asian Surinamese, African Surinamese, Ghanaian, Turkish, and Moroccan descent living in Amsterdam and were randomly sampled from the municipality register. Nineteen FHL2 polymorphisms were genotyped, and associations with lipid panels and T2D status were investigated. We observed that seven FHL2 polymorphisms associated nominally with a pro-diabetogenic lipid profile including triglyceride (TG), high-density and low-density lipoprotein-cholesterol (HDL-C and LDL-C), and total cholesterol (TC) concentrations, but not with blood glucose concentrations or T2D status in the complete HELIUS cohort upon correcting for age, gender, BMI, and ancestry. Upon stratifying for ethnicity, we observed that only two of the nominally significant associations passed multiple testing adjustments, namely, the association of rs4640402 with increased TG and rs880427 with decreased HDL-C concentrations in the Ghanaian population. Our results highlight the effect of ethnicity on pro-diabetogenic selected lipid biomarkers within the HELIUS cohort, as well as the need for more large multiethnic cohort studies.

## 1. Introduction

Type 2 diabetes mellitus (T2D) is a highly prevalent and complex metabolic disorder affecting millions of people worldwide [1]. T2D is characterized by insulin resistance accompanied by progressive pancreatic β-cell failure, leading to hyperglycemia [2]. Additionally, it is known that approximately 50% of T2D patients develop dyslipidemia, resulting in increased fasting triglycerides (TG) and low-density lipoprotein cholesterol (LDL-C) concentrations, as well as decreased high-density lipoprotein cholesterol (HDL-C) concentrations [3,4,5,6]. The combination of hyperglycemia and dyslipidemia is a strong driver of cardiovascular disease. Furthermore, while the global prevalence of T2D is on the rise, it also appears that there are differences in the risk of developing T2D, dyslipidemia, and associated cardiovascular complications across ethnic groups [7,8], which may be caused by differences in the genetic background of individuals. Indeed, several genome-wide association studies (GWASs) have linked multiple single-nucleotide polymorphisms (SNPs) to T2D [9] and dyslipidemia [10]. However, many of these GWASs have been conducted in mostly European descent populations and provide less insight into the contribution of genetics to differences in T2D and dyslipidemia across ethnic groups.

Four and a half LIM domains 2 (FHL2) is a member of the FHL domain family of proteins. FHL2 is expressed most abundantly in the heart and muscles, and to a lesser extent in other organs [11,12,13,14]. FHL2 serves as an interaction platform that acts through various protein–protein interactions [15,16]. Upon binding to a target protein, FHL2 may either enhance or repress the binding of the target protein to another protein or may alter the conformation of the target protein [17]. Through binding with a target protein, FHL2 can regulate various protein signaling pathways [15]. Thus far, FHL2 has been researched extensively in the field of oncology and cardiovascular diseases, as well as inflammation and cell differentiation, although far less is known regarding its involvement in metabolism [11,18,19,20,21]. It is only recently, however, that publications have surfaced which demonstrate a link between FHL2 and metabolism. As such, GWASs have shown an association between FHL2 loci and body mass index (BMI) [22,23]. Interestingly, studies have also implicated FHL2 in glucose metabolism and diabetes-related complications [24,25,26]. Most recently, we demonstrated that FHL2-deficient mice are protected from weight gain on a high-fat diet. These mice show increased energy expenditure involving browning of the white adipose tissue and increased glucose uptake in the heart [27]. In line with these observations, we confirmed that, in human adipose tissue, the expression of FHL2 negatively associates with the expression of browning genes. Additionally, we also showed that FHL2 expression was higher in individuals with T2D than non-diseased individuals using publicly available human pancreatic islet datasets and that FHL2-deficient mice possessed improved glucose clearance compared to wild type (WT) mice [26]. In the same pancreatic islet datasets, we also observed a correlation between higher FHL2 expression and higher HbA1c levels. The purpose of the current study was to determine whether FHL2 genetic loci are associated with the incidence of T2D and various aspects of lipid metabolism in a large multiethnic cohort (HELIUS cohort) and, thus, to further elucidate the role of FHL2 in human T2D and dyslipidemia. Here, we hypothesized that FHL2 SNPs associate with specific markers of glucose and lipid metabolism such as fasting plasma glucose values and plasma TG concentrations in humans.

## 2. Results

### 2.1. Baseline Characteristics

The cohort we analyzed in this study consisted of 10,056 subjects of both male and female gender from different ethnic backgrounds including European Dutch, African Surinamese, South Asian Surinamese, Ghanaian, Turkish, and Moroccan. The relative contribution of participants from each ethnic group was unequal in this study. In order of relative size, the largest groups were Moroccan (30.1%), Turkish (26.2%), South Asian Surinamese (14.9%), European Dutch (12.8%), African Surinamese (11.5%), and Ghanaian origin (4.4%) (Table 1). The percentage of males per ethnicity differed across groups, with the highest percentage of males being present within the European Dutch group (50%). Interestingly, we also observed differences in the percentage of T2D individuals within groups, with European Dutch participants having the lowest prevalence (5.8%) and the South Asian Surinamese group having the highest (21.4%). The baseline characteristics of the cohort varied across ethnicities. The mean age was lowest in the Turkish and Moroccan groups (41 ± 12 years and 41 ± 13 years, respectively) and highest in the European Dutch (51.8 ± 13 years) and African Surinamese (52 ± 11 years) groups. Mean BMI also differed across groups, with European Dutch participants having on average lower BMI (25.5 ± 4.4 kg/m^2^) than Turkish participants (28.5 ± 5.6 kg/m^2^). South Asian Surinamese participants showed the largest waist-to-hip ratio (WHR), while the Moroccan participants showed the smallest. Fat percentage was lowest in the European Dutch group (29.4% ± 7.5%) and highest in the Moroccan group (32.8% ± 8.3%). Fasting plasma glucose and HbA1c concentrations were lowest in the European Dutch group (5.4 ± 0.8 mmol/L and 36.9 ± 4.9 mmol/mol, respectively) and highest in the South Asian Surinamese group (5.9 ± 1.5 mmol/L and 42.6 ± 10.1 mmol/mol, respectively). Blood TG concentrations were lowest in the Ghanaian group (0.7 ± 0.4 mmol/L) and highest in the Turkish group (1.2 ± 0.9 mmol/L), while the inverse was true for blood HDL-C concentrations. TC and blood LDL-C concentrations were both highest in the European Dutch participants (5.2 ± 1.0 mmol/L and 3.2 ± 0.9 mmol/L) and lowest in the Moroccan participants (4.6 ± 0.9 mmol/L and 2.9 ± 0.8 mmol/L), respectively (Table 1).

### 2.2. FHL2 Genetic Polymorphism Distribution

**Figure 1:**
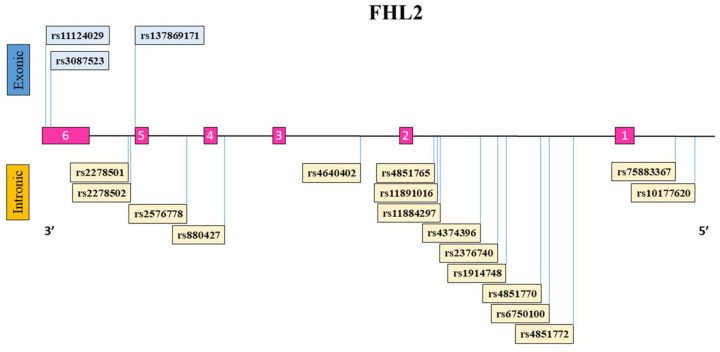
Schematic overview of the FHL2 gene and the genetic polymorphisms studied here. The FHL2 reference sequence gene consists of six exons (numbered 1–6 in pink) and five introns. FHL2 SNPs are indicated in boxes and their location on the gene is depicted with a line. FHL2 SNPs listed above the FHL2 gene are located within exonic regions (blue), while SNPs listed at the bottom of the gene are present within intronic/intergenic regions of the gene (yellow).

A schematic representation of the FHL2 gene with the exons (1–6) and introns along with the location of each FHL2 SNP is illustrated (Figure 1). Additionally, the FHL2 polymorphisms with their respective reference and alternative alleles, position within the genome, SNP type classification, and ethnicity-specific allele frequency in this study are indicated (Table 2). The distribution of FHL2 SNP reference allele and alternative allele among the different ethnicities differed substantially in some cases. The Ghanaian subjects demonstrated the highest prevalence of the reference alleles for SNPs rs11124029, rs3087523, rs2278502, rs257678, rs880427, rs2376740, rs4851770, and rs7583367. In contrast, the Ghanaian group also showed the lowest proportion of the reference allele for SNP rs2278501, rs4640402, and rs6750100 compared to the other ethnicities. The alternative allele for the missense SNP rs137869171 leading to an Asn226Lys amino-acid change was only present within the European Dutch and Moroccan groups. Of the 19 FHL2 genetic polymorphisms that we evaluated, rs11124029 and rs3087523 lead to synonymous polymorphisms which do not alter the amino-acid sequence of the resulting protein.

### 2.3. Associations between FHL2 SNPs and Lipid Metabolism and Glucose Tolerance

**Figure 2:**
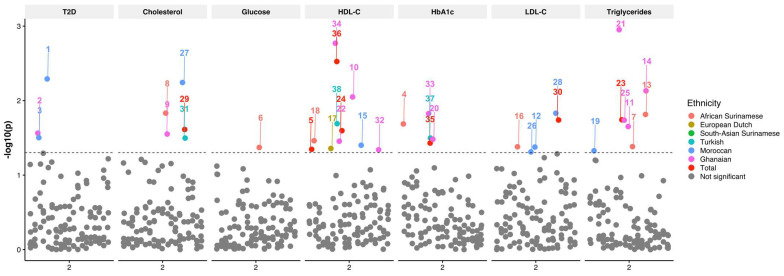
Manhattan plot of FHL2 SNPs associated with T2D, total cholesterol, glucose, HDL-C, HbA1c, LDL-C, and triglycerides. The X-axis depicts the chromosome. The Y-axis depicts the −log_10_(p) of the corresponding *p*-value. The different colors indicate the results per group with African Surinamese (orange), European Dutch (macaroon), South Asian Surinamese (green), Turkish (blue), Moroccan (dark blue), Ghanaian (purple), total cohort (red), and not significant (gray).

Univariate analysis of FHL2 SNPs with age, gender, BMI, and ancestry as covariates showed nominally significant associations between FHL2 SNPs alternative alleles and plasma TG, HDL-C, LDL-C, and TC concentrations (Figure 2). The rs11124029 SNP was associated with a decreased HDL-C concentration (*p* = 0.045, beta = −0.009). The SNP rs4640402 was associated with a decreased TG concentration (*p* = 0.018, beta = −0.017), whereas it was associated with an increased HDL-C concentration (*p* = 0.025, beta = 0.008). On the other hand, the rs880427 SNP was associated with a decreased HDL-C concentration (*p* = 0.003, beta = −0.011), as well as increased HbA1c (*p* = 0.037, beta = 0.004). Furthermore, the SNP rs4851770 was associated with both an increased LDL-C (*p* = 0.018, beta = 0.01) and TC concentration (*p* = 0.024, beta = 0.006). In addition to our analysis of the complete HELIUS cohort, the multiethnic composition allowed us to evaluate whether the SNP association with the outcomes were similar across ethnic groups. To this end, we stratified our association analysis by ethnicity and conducted the same test with the same covariates in each subset. In the ethnicity-stratified analyses, we saw 31 nominally significant associations. We saw the most associations in the Moroccan group, where two different SNPs were associated with a decreased LDL-C concentration (rs11891016 and rs4851765), and the SNP rs4851770 was associated with an increased LDL-C concentration (*p* = 0.015, beta = 0.018). This SNP was also associated with an increased TC concentration (*p* = 0.006, beta = 0.014). In this group, the SNPs rs137869171 (*p* = 0.005, OR = 3.757) and rs2278501 (*p* = 0.031, OR = 1.224) were associated with an increased risk of T2D. In this group, we additionally saw that the SNP rs3087523 was associated with a decreased TG concentration (*p* = 0.047, beta = −0.047). Lastly, we also observed that rs2376740 was associated with a decrease in HDL-C concentration (*p* = 0.04, beta = −0.013).

Interestingly, in the African Surinamese group, the SNP rs3087523 was associated with decreased HDL-C (*p* = 0.035, beta = −0.067), and the SNPs rs1914748 and rs11884297 were associated with decreased TG concentrations (*p* = 0.047, beta = −0.047 and *p* = 0.041, beta = −0.044). Furthermore, rs2576778 was associated with an increase in LDL-C (*p* = 0.042, beta = 0.045). Additionally, the SNPs rs11124029 (*p* = 0.021, beta = 0.025) were associated with increased HbA1c. Lastly, rs118884297 was associated with a decrease in TC (*p* = 0.015, beta = −0.023), as well as plasma glucose (*p* = 0.043, beta = −0.021). In the Ghanaian group, we found that the SNP rs2278501 was associated with an increased risk of T2D (*p* = 0.027, OR = 1.61), and the SNP rs880427 was associated with a decreased HDL-C concentration (*p* = 0.002, beta = −0.071). On the other hand, the rs11884297 SNP was associated with an increased HDL-C concentration (*p* = 0.009, beta = 0.053) and an increased TC concentration (*p* = 0.028, beta = 0.036). Moreover, the rs4640402 SNP was associated with a decreased HDL-C concentration (*p* = 0.035, beta = −0.037), an increased TG concentration (*p* = 0.001, beta = 0.103), and an increased HbA1c concentration (*p* = 0.033, beta = 0.028). Three SNPs were associated with a decreased TG concentration (rs11891016: *p* = 0.02, beta = −0.08; rs1914748: *p* = 0.007, beta = −0.08; rs4851765: *p* = 0.018, beta = −0.09). Lastly, the SNP rs48511772 was associated with a decrease in HDL-C (*p* = 0.046, beta = −0.04), and rs880427 was associated with an increase in HbA1c (*p* = 0.015, beta = 0.04).

In the Turkish group, the SNP rs4851770 was associated with an increased TC concentration (*p* = 0.038, beta = 0.011). Furthermore, the rs880427 SNP was associated with a decreased HDL-C concentration (*p* = 0.02, beta = −0.016) and increased HbA1c (*p* = 0.032, beta = −0.008). In the European Dutch group, SNP rs2576778 was associated with an increase in HDL-C concentration (*p* = 0.044, beta = 0.024). Only two of the nominally significant associations passed multiple testing adjustments, namely, the association of the SNP rs4640402 (*p* = 0.002, beta = 0.103) with increased TG and the association of the rs880427 (*p* = 0.003, beta = 0.07) with decreased HDL-C concentrations in the Ghanaian population. All FHL2 SNP associations listed here are indicated in Table 3 and Supplementary File S1.

## 3. Discussion

In this study, we elucidated the associations between several FHL2 SNPs and multiple parameters of lipid metabolism including TG, HDL-C, LDL-C, and TC, as well as T2D status, HbA1c, and glucose concentrations, in the HELIUS cohort. In addition, this is one of the first studies to make use of the genotype data available from the HELIUS cohort and investigate the novel metabolism-related gene FHL2 and its polymorphisms in a multiethnic setting. In doing so, we illustrate for the first time the association of several FHL2 polymorphisms with plasma lipid concentrations and hyperglycemia. We also demonstrate that there appears to exist not only concordant but also opposing associations of these SNPs with outcomes between ethnic groups.

We identified the SNP rs4851770 to be associated with increased LDL-C and TC concentrations in the complete cohort, as well as in the Turkish group, and with increased TC concentration in the Moroccan group. The SNP rs2278501 was associated with an increased risk of T2D in the Ghanaian and Moroccan groups. Lastly, rs880427 was associated with a decreased HDL-C concentration in the total cohort and in the Ghanaian and Turkish groups.

On the other hand, we also identified seemingly opposing effects. The SNP rs3087523 SNP was associated with a decreased HDL-C concentration in the African Surinamese, but with a decreased TG concentration in the Moroccan group. The power of our associations varied greatly, from 5% to 89%, with a mean power of 53%. If considering all association tests, the power varied from 5% to 89%, with a mean power of only 12%. This is likely a reflection of the still rather small sample size in this cohort and suggests that, in addition to environmental factors, multiple SNPs may be involved in driving these phenotypes. Thus, a validation study in a larger sample size could elucidate whether these associations are robust. Furthermore, while various lipid measurements were performed in this cohort, these were by no means exhaustive and did not include, for example, ceramides or plasmalogens. Subsequent studies in large multiethnic cohorts will benefit greatly from including a more exhaustive lipid panel.

Both T2D and dyslipidemia are complex metabolic disorders that affect large portions of the global population and are associated not only with one another, but also with other metabolic diseases. In this study, we aimed to uncover the link between FHL2 genetic polymorphisms and dyslipidemia, as well as T2D, using the large multiethnic HELIUS cohort. FHL2 is still a relatively unknown gene in the field of metabolism with currently only a handful of publications. Given that FHL2 has been mechanistically associated with insulin secretion [24,26], diabetic kidney disease [25], and obesity [27], and that SNPs and epigenetic changes in FHL2 are associated with T2D [24] and body fat mass [28], we hypothesized that FHL2 genetic variants may also be associated with specific markers of glucose and lipid metabolism such as fasting plasma glucose values and plasma TG concentrations in humans.

We focused on T2D-related parameters such as plasma glucose and HbA1c concentrations as previous work by our group showed a correlation between FHL2 expression and HbA1c levels [26]. While we did not observe any significant associations between the FHL2 SNP variants and plasma glucose concentrations, we did uncover nominally significant associations with blood TG, HDL-C, LDL-C, and TC concentrations, as well as with T2D status and HbA1c concentration. FHL2 SNPs were associated with a pro-diabetogenic lipid profile with elevated LDL-C and TC, as well as decreased HDL-C. However, some FHL2 SNPs were also associated with increased HDL-C and decreased TG. This is interesting as we recently elucidated the protective role of FHL2 deficiency against developing obesity in mice and highlighted the association between FHL2 expression and browning of white adipose tissue in humans [27]. Adipocytes also regulate serum TG and HDL-C. Considering that FHL2 expression plays a role in adipocyte phenotype in mice and potentially in humans, and that adipocytes regulate serum TG and HDL-C, genetic variants in the FHL2 gene may also affect blood TG and HDL-C concentrations in humans.

Of the 19 FHL2 genetic polymorphisms that we evaluated, rs11124029 and rs3087523 lead to synonymous polymorphisms which do not alter the amino-acid sequence of the resulting FHL2 protein, while rs137869171 does lead to a missense polymorphism that alters the amino-acid sequence of FHL2 (Asn226Lys). FHL2 is composed of nine zinc fingers, and this variation is located in the eighth zinc finger, changing a polar uncharged amino acid into a positively charged amino acid, which may have functional consequences for the protein that are at present unknown. In our analyses, however, we only observed a nominally significant association between rs137869171 and an increased risk of T2D in the Moroccan group. The remaining FHL2 genetic loci were located in noncoding regions such as introns and intergenic regions upstream of FHL2.

Our results showed that only two of the nominally significant associations passed multiple testing adjustments, namely, the association of SNP rs4640402 with increased TG and the association of SNP rs880427 with decreased HDL-C concentrations in the Ghanaian population. These SNPs highlight the potential contribution of FHL2 to the risk of developing pro-diabetogenic lipid profile in this group. Our results within the Ghanaian group present similarities with previous work, which demonstrated the impact of a pro-diabetogenic polymorphisms in Japanese men associated with increased susceptibility to T2D [29]. However, whether the FHL2 SNP associations we highlight here in the Ghanaian group are truly causal requires further inquiry. The genetic variation within intronic and intergenic regions may still have functional implications for FHL2 expression through the regulation of alternative splicing, in addition to affecting promoter and enhancer regions upstream of FHL2. In addition, it has also been demonstrated that synonymous polymorphisms may elicit non-neutral effects in mRNA gene expression and, thus, negatively impact the organism in which they occur [30]. However, this would still need to be studied in further detail and is currently beyond the scope of this study.

In conclusion, our data indicate a link between FHL2 polymorphisms and dyslipidemia that is dependent on ethnic differences between individuals but does not occur through an effect on glucose metabolism. This was most clearly visible in the Ghanaian group after correcting for multiple testing. Given the vast array of targets that FHL2 can bind to, as well as recent publications demonstrating its role in metabolism, it stands to reason that we do not yet fully understand the role of FHL2 in metabolism or the underlying mechanisms such as genetic variation that determine its expression and function.

## 4. Materials and Methods

### 4.1. Population

The Healthy Life in an Urban Setting (HELIUS) study is a large multiethnic cohort study conducted in Amsterdam, the Netherlands, from which data was collected from January 2011 to November 2015; this study was described in detail elsewhere [31,32]. Briefly, the cohort contains individuals of European Dutch, South Asian Surinamese, African Surinamese, Ghanaian, Turkish, and Moroccan descent ranging from 18 to 70 years old, living in/near Amsterdam. Potential participants were sampled with a simple random sampling method from the municipality registry, after stratification by ethnicity as defined by registered country of birth.

The complete study population consisted of 24,789 participants of European Dutch (n = 4671), South Asian Surinamese (n = 3369), African Surinamese (n = 4458), Ghanaian (n = 2735) Turkish (n = 4200), Moroccan descent (n = 4502), and unknown Surinamese or unknown descent (n = 854), of which a subset had whole-genome genotyping data used for further analysis in this study [28,31]. Specifically, we analyzed the data of 10,056 individuals from the subset of the HELIUS cohort with genotyping data from the six largest ethnic groups, which equated to 1286 European Dutch, 1502 South Asian Surinamese, 1156 African Surinamese, 445 Ghanaian, 2636 Turkish, and 3031 Moroccan. In the total results, all subjects were included.

The study protocols were previously approved by the Amsterdam Medical Center ethical review board, and all participants provided written and informed consent. Ethnicity was defined by the country of birth of the participants, as well as that of their parents. The exact distinction of ethnicity in the HELIUS cohort was also described more extensively elsewhere [31]. Briefly, subjects were classified as European Dutch if they were born in the Netherlands and if both parents were also of European Dutch origins. All non-European Dutch participants in this study were classified on the basis of whether they were born outside of the Netherlands and had at least one parent who was also born outside of the Netherlands, or they were born in the Netherlands but both parents were born elsewhere. A limitation of the country of birth indicator for ethnicity is that people who were born in the same country might have a different ethnic background, which, in the Dutch context, applies to the Surinamese population (Table 1). Therefore, after data collection, participants of Surinamese ethnic origin were further classified according to self-reported ethnic origin (obtained by questionnaire) into ‘African’, ‘South-Asian’, ‘Javanese’, or ‘other’. The homogeneity of each ethnic group was demonstrated previously for the genome [33], microbiome [34], and diet [35].

### 4.2. Phenotypical Assessments

Participants completed a structured questionnaire with records on demographic, socioeconomic, and health-related behavior. Height measurement was performed without shoes with SECA 217 stadiometer to the nearest 0.1 cm. Weight was measured without shoes and in light clothing with SECA 877 scales to the nearest 0.1 kg. Body mass index (BMI) was determined by dividing measured body weight (kg) by height squared (m^2^). Fasting blood samples were drawn, and plasma samples were used to determine the concentration of glucose by spectrophotometry, using hexokinase as the primary enzyme (Roche Diagnostics, Tokyo, Japan). In this study, we defined individuals suffering from T2D according to whether they self-reported as such, had increased fasting glucose (≥7 mmol/L), or used glucose-lowering medication. Blood samples were drawn from all participants in a fasted state (>8 h of fasting). Serum TG, total cholesterol (TC), HDL-cholesterol (HDL-C), glucose, and LDL-cholesterol (LDL-C) concentrations were measured/calculated from plasma samples, while whole blood was used to determine hemoglobin A1C (HbA1c) concentrations as described previously, using an in-house assay [36]. The continuous measurements were log_10_-transformed prior to association testing.

### 4.3. Genotyping and Polymorphism Quality Control

Genotyping of HELIUS participants was performed as described elsewhere [37,38]. After the original quality control, the autosomal chromosomes were imputed using the Sanger Imputation Service (https://imputation.sanger.ac.uk, accessed on 1 May 2021). Phasing was performed with EAGLE2 and the PBWT method using the HAPLOTYPE Reference Consortium (release 1.1). Thereafter, poorly imputed SNPs were filtered using a 0.5 imputation quality score cutoff. All chromosome locations are based on the GRCh37 coordinates. Additional quality control was performed using PLINK version 1.9 (the following parameters for quality tests were used: --geno 0.05 --mind 0.05 --indep-pairwise 50 5 0.5 --genome --min 0.1875 --hwe 0.00001). The following SNPs were directly genotyped on the array, whereas the rest were imputed: rs880427, rs1914748, and rs6750100. In the full cohort, SNP variant rs137869171 had a minor allele frequency (MAF) <1%, while variants rs2278502, rs2376740, and rs11891016 were in linkage disequilibrium. Despite this, all FHL2 genetic polymorphisms were used for further analysis. No quality issues were observed for any of the participants. All FHL2 SNP variants underwent quality control per ethnic group for the ethnicity-specific analyses. For the European Dutch group, all variants passed the MAF threshold of 1% with rs2278502, rs2376740, and rs11891016 being in LD. In the South Asian Surinamese group, all variants passed MAF 1% with only rs2278502 and rs11891016 being in LD. All variants in the African Surinamese group passed MAF 1%. In the Ghanaian group, rs137869171 did not meet the criteria of MAF 1% and was in LD alongside rs11891016. For the Turkish group, all FHL2 SNP variants met the MAF 1% criteria while rs2278502, rs11891016, and rs2376740 were in LD. Lastly, in the Moroccan group, all SNP variants met the criteria of MAF 1%; however, the variants rs2278502, rs2376740, rs11891016, and rs11124029 were in LD.

## Figures and Tables

**Table 1:** Baseline characteristics of the HELIUS cohort stratified by ethnicity.

Baseline Characteristics	European Dutch	South Asian Surinamese	African Surinamese	Ghanaian	Turkish	Moroccan
No. of participants	1286	1502	1156	445	2636	3031
Total cohort (%)	12.8	14.9	11.5	4.4	26.2	30.1
Males (% of ethnicity)	50	46	39	41	46	39
Diabetic individuals (% of ethnicity)	5.8	21.4	15.4	14.6	10.2	11.7
Age (years)	51.8 ± 13	47.0 ± 13	52 ± 11	48 ± 9	41 ± 12	41 ± 13
BMI (kg/m^2^)	25.5 ± 4.4	26.4 ± 4.6	28.4 ± 5.5	28.4 ± 4.6	28.5 ± 5.6	27.7 ± 5.2
Waist-to-hip ratio (WHR)	0.9 ± 0.1	0.9 ± 0.1	0.9 ± 0.1	0.9 ± 0.1	0.9 ± 0.1	0.9 ± 0.1
BIA Fat percentage (%)	29.4 ± 7.5	32.0 ± 8.2	32.7 ± 9.0	32.6 ± 8.9	31.9 ± 8.3	32.8 ±8.3
Fasting blood glucose (mmol/L)	5.4 ± 0.8	5.9 ± 1.5	5.6 ± 1.3	5.5 ± 1.4	5.5 ± 1.2	5.5 ± 1.4
Fasting blood HbA1c (mmol/mol)	36.9 ± 4.9	42.6 ± 10.1	40.7 ± 9.1	40.2 ± 11.0	38.7 ± 8.3	38.9 ± 8.7
Blood triglyceride (mmol/L)	1.1 ± 0.7	1.2 ± 0.9	0.9 ± 0.6	0.7 ± 0.4	1.2 ± 0.9	1.0 ± 0.6
Blood total cholesterol (mmol/L)	5.2 ± 1.0	5.0 ± 1.1	5.0 ± 1.0	5.0 ± 1.0	4.9 ± 1.0	4.6 ± 0.9
Blood HDL (mmol/L)	1.5 ± 0.4	1.3 ± 0.4	1.5 ± 0.4	1.6 ± 0.4	1.3 ± 0.4	1.3 ± 0.3
Blood LDL (mmol/L)	3.2 ± 0.9	3.1 ± 0.9	3.0 ± 0.9	3.0 ± 0.9	3.0 ± 0.8	2.9 ± 0.8

Results are indicated as the mean and standard deviation or as percentages of either total cohort or specified ethnicity.

**Table 2:** Distribution of FHL2 SNPs per ethnicity.

FHL2 SNP				European Dutch		South Asian Surinamese		African Surinamese		Ghanaian		Turkish		Moroccan	
SNP ID	Ref Allele	Alt Allele	Type	Ref Allele	Alt Allele	Ref Allele	Alt Allele	REF ALLELE	Alt Allele	Ref Allele	Alt Allele	Ref Allele	Alt Allele	Ref Allele	Alt Allele
rs11124029	G	A	Synonymous	0.79	0.21	0.88	0.12	0.91	0.09	0.92	0.08	0.79	0.21	0.83	0.17
rs3087523	G	A	Synonymous	0.89	0.11	0.96	0.04	0.97	0.03	0.98	0.02	0.91	0.09	0.93	0.07
rs2278501	T	C	Intron	0.54	0.46	0.47	0.53	0.31	0.69	0.28	0.72	0.49	0.51	0.35	0.65
rs2278502	C	A	Intron	0.61	0.39	0.64	0.36	0.8	0.2	0.86	0.14	0.63	0.37	0.74	0.26
rs137869171	G	A	Missense	0.99	0.01	1	0	1	0	1	0	1	0	0.99	0.01
rs2576778	G	A	Intron	0.8	0.2	0.78	0.22	0.9	0.1	0.91	0.09	0.83	0.17	0.9	0.1
rs880427	G	A	Intron	0.66	0.34	0.65	0.35	0.81	0.19	0.84	0.16	0.64	0.36	0.68	0.32
rs4640402	A	C	Intron	0.59	0.41	0.45	0.55	0.38	0.62	0.34	0.66	0.6	0.4	0.61	0.39
rs4851765	T	C	Intron	0.66	0.34	0.64	0.36	0.76	0.24	0.8	0.2	0.67	0.33	0.8	0.2
rs11891016	C	T	Intron	0.67	0.33	0.64	0.36	0.74	0.26	0.78	0.22	0.68	0.32	0.81	0.19
rs11884297	C	T	Intron	0.57	0.43	0.68	0.32	0.72	0.28	0.79	0.21	0.61	0.39	0.62	0.38
rs4374396	A	G	Intron	0.64	0.36	0.58	0.42	0.62	0.38	0.6	0.4	0.63	0.37	0.58	0.42
rs2376740	C	T	Intron	0.57	0.43	0.7	0.3	0.69	0.31	0.78	0.22	0.58	0.42	0.62	0.38
rs1914748	C	T	Intron	0.54	0.46	0.49	0.51	0.51	0.49	0.44	0.56	0.53	0.47	0.5	0.5
rs4851770	C	T	Intron	0.5	0.5	0.58	0.42	0.74	0.26	0.84	0.16	0.53	0.47	0.61	0.39
rs6750100	A	G	Intron	0.75	0.25	0.69	0.31	0.61	0.39	0.53	0.47	0.76	0.24	0.72	0.28
rs4851772	A	G	Intron	0.92	0.08	0.82	0.18	0.77	0.23	0.73	0.27	0.85	0.15	0.77	0.23
rs7583367	G	T	Intergenic	0.53	0.47	0.61	0.39	0.82	0.18	0.92	0.08	0.55	0.45	0.65	0.35
rs10177620	A	G	Intergenic	0.67	0.33	0.66	0.34	0.6	0.4	0.56	0.44	0.7	0.3	0.67	0.33

FHL2 allele distribution per SNP for reference allele and alternative allele stratified per ethnicity. The exact position and SNP classification type are based on the most recent version of the human reference genome version 37 (hg37). Allele frequencies are calculated on the basis of prevalence within the HELIUS dataset. Reference allele frequencies in the Ghanaian group were the highest/lowest in the HELIUS cohort in 16/19 FHL2 SNPs.

**Table 3:** Summary results of FHL2 SNPS associations in HELIUS cohort.

Outcome	SNP ID	Ethnicity	Raw *p*-Value
HbA1c	rs11124029	African Surinamese	2.06 × 10^−2^
Glucose	rs11884297	African Surinamese	4.25 × 10^−2^
Triglycerides	rs11884297	African Surinamese	4.14 × 10^−2^
Cholesterol	rs11884297	African Surinamese	1.47 × 10^−2^
Triglycerides	rs1914748	African Surinamese	1.53 × 10^−2^
LDL-C	rs2576778	African Surinamese	4.17 × 10^−2^
HDL-C	rs3087523	African Surinamese	3.45 × 10^−2^
HDL-C	rs2576778	European Dutch	4.39 × 10^−2^
T2D	rs2278501	Ghanaian	2.73 × 10^−2^
Cholesterol	rs11884297	Ghanaian	2.81 × 10^−2^
HDL-C	rs11884297	Ghanaian	8.92 × 10^−3^
Triglycerides	rs11891016	Ghanaian	2.23 × 10^−2^
Triglycerides	rs1914748	Ghanaian	7.39 × 10^−3^
HbA1c	rs4640402	Ghanaian	3.30 × 10^−2^
Triglycerides	rs4640402	Ghanaian	1.12 × 10^−3^
HDL-C	rs4640402	Ghanaian	3.51 × 10^−2^
Triglycerides	rs4851765	Ghanaian	1.83 × 10^−2^
HDL-C	rs4851772	Ghanaian	4.57 × 10^−2^
HbA1c	rs880427	Ghanaian	1.49 × 10^−2^
HDL-C	rs880427	Ghanaian	1.70 × 10^−3^
T2D	rs137869171	Moroccan	5.11 × 10^−3^
T2D	rs2278501	Moroccan	3.14 × 10^−2^
LDL-C	rs11891016	Moroccan	4.20 × 10^−2^
HDL-C	rs2376740	Moroccan	3.97 × 10^−2^
Triglycerides	rs3087523	Moroccan	4.71 × 10^−2^
LDL-C	rs4851765	Moroccan	4.88 × 10^−2^
Cholesterol	rs4851770	Moroccan	5.71 × 10^−3^
LDL-C	rs4851770	Moroccan	1.48 × 10^−2^
HDL-C	rs11124029	Total	4.51 × 10^−2^
Triglycerides	rs4640402	Total	1.81 × 10^−2^
HDL-C	rs4640402	Total	2.53 × 10^−2^
Cholesterol	rs4851770	Total	2.44 × 10^−2^
LDL-C	rs4851770	Total	1.82 × 10^−2^
HbA1c	rs880427	Total	3.71 × 10^−2^
HDL-C	rs880427	Total	2.99 × 10^−3^
Cholesterol	rs4851770	Turkish	3.18 × 10^−2^
HbA1c	rs880427	Turkish	3.18 × 10^−2^
HDL-C	rs880427	Turkish	2.04 × 10^−2^

FHL2 SNP associations with multiple parameters in total HELIUS cohort (Total) and per ethnicity. Results are sorted in alphabetical order on the basis of ethnicity, and raw *p*-values are indicated in the rightmost column.

## Data Availability

The HELIUS data are owned by the Amsterdam University Medical Centers, located at the AMC in Amsterdam, The Netherlands. The data can be requested by submitting a proposal to the HELIUS Executive Board as detailed at http://www.heliusstudy.nl/en/researchers/collaboration, accessed on 1 May 2021, by email: heliuscoordinator@amsterdamumc.nl. The HELIUS Executive Board will check proposals for compatibility with the general objectives, ethical approval and informed consent forms of the HELIUS study. All requests regarding HELIUS data will be processed in the same manner.

## Supplementary Materials

The following supporting information can be downloaded at https://www.mdpi.com/article/10.3390/ijms24054332/s1.
